# The Impact of Sphingosine Kinase-1 in Head and Neck Cancer

**DOI:** 10.3390/biom3030481

**Published:** 2013-08-12

**Authors:** Paulette M. Tamashiro, Hideki Furuya, Yoshiko Shimizu, Kayoko Iino, Toshihiko Kawamori

**Affiliations:** 1 Cancer Biology Program, University of Hawaii Cancer Center, 701 Ilalo Street, Honolulu, HI 96813, USA; E-Mails: pyamada@cc.hawaii.edu (P.M.T.); hfuruya@cc.hawaii.edu (H.F.); yshimizu@cc.hawaii.edu (Y.S.); kiino@cc.hawaii.edu (K.I.); 2 Department of Molecular Biosciences and Bioengineering, University of Hawaii at Manoa, Honolulu, HI 96818, USA; E-Mail: yshimizu@cc.hawaii.edu

**Keywords:** sphingolipids, SphK1, S1P, head and neck cancer, invasion and proliferation

## Abstract

Head and neck squamous cell carcinoma (HNSCC) has a high reoccurrence rate and an extremely low survival rate. There is limited availability of effective therapies to reduce the rate of recurrence, resulting in high morbidity and mortality of advanced cases. Late presentation, delay in detection of lesions, and a high rate of metastasis make HNSCC a devastating disease. This review offers insight into the role of sphingosine kinase-1 (SphK1), a key enzyme in sphingolipid metabolism, in HNSCC. Sphingolipids not only play a structural role in cellular membranes, but also modulate cell signal transduction pathways to influence biological outcomes such as senescence, differentiation, apoptosis, migration, proliferation, and angiogenesis. SphK1 is a critical regulator of the delicate balance between proliferation and apoptosis. The highest expression of SphK1 is found in the advanced stage of disease, and there is a positive correlation between SphK1 expression and recurrent tumors. On the other hand, silencing SphK1 reduces HNSCC tumor growth and sensitizes tumors to radiation-induced death. Thus, SphK1 plays an important and influential role in determining HNSCC proliferation and metastasis. We discuss roles of SphK1 and other sphingolipids in HNSCC development and therapeutic strategies against HNSCC.

## 1. Introduction

Recurrence rates for advanced-stage head and neck squamous cell carcinoma (HNSCC) is greater than 50% [1]. In addition, the five-year survival rate for HNSCC is 50%, and has not drastically improved over the last 30 years [2]. This may be due to late presentation and the subsequent delay in detection of lesions, and a high rate of metastasis and invasion into locoregional lymph nodes [2]. Furthermore, there is limited availability of effective therapies to reduce the rate of recurrence, resulting in high morbidity and mortality of advanced cases [2]. Therefore, the purpose of this review is to provide an overview of the effect of sphingosine kinase-1 (SphK1) on HNSCC and offer insight into possible uses of SphK1 levels affect other sphingolipid metabolites and enzymes, this review also summarizes the contributions of other sphingolipids to HNSCC etiology.

## 2. Sphingolipid Overview

Sphingolipids are a family of lipids that largely exist in cellular membranes to provide structural support, mechanical stability, a protective barrier, adhesion sites for extracellular proteins, subdomain structure of microdomains (lipid rafts), and regulate caveolar-mediated endocytosis [3,4,5]. In addition to their structural role, they modulate cell signal transduction pathways to influence cell physiology and biological outcomes such as cell senescence, differentiation, apoptosis, cell-cell arrest, migration, proliferation, angiogenesis, and inflammation [3,6]. To date sphingosine, ceramide, ceramide-1-phosphate (C1P), glycosylceramide, lyso-sphingomyelin, and dihydroceramide and sphingosine 1-phosphate (S1P) have been identified as bioactive lipids, each exerting distinct effects on proliferation. Sphingolipid-related pathways in cancer have been eloquently described previously and should be referred to for a more comprehensive review [3,6].

The multi-faceted roles of sphingolipids make the study of their regulation complex. Firstly, sphingolipids are interconnected in a sensitive network, where increased enzymatic activity can convert pro-apoptotic ceramide through sphingosine to pro-survival S1P and shift the balance to cell survival, migration and inflammation [7]. In a converse scenario, ceramide can be hydrolyzed to sphingosine resulting in senescence and apoptosis [8]. Thus, it is important to keep in mind that although it is possible that increases or decreases in particular enzymes or substrates may coincide with biological effects, the particular enzyme or substrate of interest may not be the true effector [6]. Along the same lines, down-regulation of a substrate or enzyme may result in compensation of another substrate or enzyme; this needs to be taken into consideration when modulating substrate and enzyme levels in sphingolipid metabolism. Given the delicate biochemical balance of each sphingolipid, without the measurement of every player in sphingolipid metabolism, it is difficult to determine the bottom line and identify the actual effector [3,6]. 

Secondly, there are multiple isoforms of sphingolipid metabolic enzymes. For example, there are two isoforms of SphK that have been cloned and characterized, SphK1 and sphingosine kinase-2 (SphK2) [9,10]. While the importance of SphK2 in HNSCC is unknown, SphK1 has been implicated in tumor growth and cell transformation in HNSCC [11]. Also, there are six ceramide synthases that catalyze the formation of ceramide, termed CerS1 through CerS6 (or LASS1 through LASS6). CerS1 and CerS6 are differentially regulated in HNSCC, indicating each enzyme has a distinct role in carcinogenesis [12].

Thirdly, S1P acts through both autocrine and intracellular signaling pathways. S1P generated through SphK1 at the plasma membrane and S1P generated by SphK2 in the nucleus and mitochondria can direct intracellular signaling (reviewed in [13]). In addition to regulation of intercellular signaling cascades, S1P is released from the cell to act on its own cell surface receptors in an autocrine manner, termed “inside-out signaling” (reviewed in [13]). S1P has five different receptors, and S1P binding to each of these receptors activates different pathways and therefore the effects of S1P appear to be cell and environment specific [14]. To matters more complex, the level of S1P and its receptors cannot in itself determine whether proliferation or apoptosis will ensue, as S1PR may be up- or down-regulated in response to the tumor microenvironment in an effort to preserve its (proliferative) action [15]. 

Lastly, sphingolipids are not centralized in one area of the cell and localize to different parts of the cell. They are synthesized in the endoplasmic reticulum and Golgi and they can travel between organelles using various transporters (reviewed in [4]). Furthermore, sphingosine kinase 1 (SphK1) resides in the cytosol, but translocates to the plasma membrane lipid raft microdomain where it is relocated in close proximity to its substrate, sphingosine. SphK1 is then able to convert sphingosine to S1P [16]. This translocation of SphK1 itself requires ligand-dependent activation of PKC and ERK, where ERK putatively phosphorylates SphK1directly at residue Ser225 to initiate translocation [17]. In another example, glucosylceramide synthase (GCS), the enzyme that converts ceramide to glucosylceramide (GluCer), is localized in the Golgi, and its sequestration in the Golgi may prevent it from acting on ceramide generated in other cell compartments [18]. Thus, intracellular locations play a role in determining the efficacy of biochemical and signal transduction control of sphingolipids. Given the characteristics and complexity of the roles of sphingolipids [3,6,13], it is important to assess multiple scenarios and rule out potential explanations for experimental outcomes before definitive conclusions are drawn. 

## 3. Head and Neck Cancer Background

Most head and neck cancers develop from the mucosal lining of the nasal cavity, paranasal sinus, larynx, trachea, oral cavity (tongue, floor or roof of the mouth, cheek lining, and gums), lip, and the naso-, hypo-, and oro-pharynx. 90% of head and neck cancers originate in the squamous cells of the epithelium of the head and neck and are referred to as squamous cell carcinoma (SCC) [19]. Esophageal cancer is also a type of SCC or adenocarcinoma, where the adenocarinoma is derived from epithelium originating from glandular tissues. HNSCC account for the majority of head and neck cancers, therefore, this paper focuses on studies utilizing these models. However, reports studying SphK1 in thyroid cancer provide valuable information and will be discussed when relevant. Thyroid cancer is categorized as a type of head and neck cancer, but differs pathologically from HNSCC.

In 2002, oral and pharyngeal cancer accounted for about 485,000 cases [20] and about 261,784 cancer-related mortalities worldwide, with males accounting for 50%–66% of both incidence and mortality cases [20]. In 2010, in the United States alone, there were about 36,540 new cases of oral or pharyngeal cancer. According to 2012 statistics, the number of cases has increased to 40,250 new cases [21]. The 2010 US statistics show that there have been about 10,990, 10,840, and 12,660 new tongue, mouth, and pharynx cancer cases, respectively [22]. Consistent with worldwide statistics, the majority of US oral cancer cases occur in males. 

Risk factors for HNSCC include tobacco and betel nut use [23], excessive alcohol consumption [23], occupational exposure such as radiation, diesel exhaust, metal, and cement dust, [24], low fruit/vegetable intake [24], gastroesophageal reflux [24], genetic susceptibility (*i.e.*, Bloom syndrome) [24], family history of HNSCC [25], poor oral hygiene [25], and human papillomavirus (HPV) [25]. Recently, HPV has received a lot of attention as a risk factor for HNSCC. A study conducted in 2007 showed that oropharyngeal cancer was significantly associated with oral HPV type 16, and seropositivity for the HPV-16-L1 capsid protein, which is a validated measure of lifetime HPV-16 exposure. Interestingly, the association between HPV-16 and oropharyngeal cancer occurred regardless of tobacco and alcohol use history, indicating that HPV is a strong factor in determining HNSCC risk. The prevalence of oral cancer is high in developing countries [20], with growing occurrence in Western countries due to its association with HPV [26].

To provide greater understanding of the genetic origins of HNSCC, whole-exome sequencing was performed on 32 [27] and 72 primary tumors [28]. Interestingly, exome sequencing revealed greater mutations in HPV-negative samples (compared to HPV-positive tumors) and tumor samples from patients that had a history of tobacco use (compared to samples obtained from patients with no history of tobacco use) [27]. In addition to the mutated genes previously implicated in HNSCC (TP53, CDKN2A, PTEN, PIK3CA, HRAS) [29,30], sequencing revealed mutations in additional genes, namely NOTCH1 [27,28], IRF6 [28], TP63 [28], and FBXW7 [27]. Since NOTCH1, IRF6, and TP63 are functionally implicated in squamous differentiation, it is plausible that these gene mutations disrupt stratified squamous differentiation and development in precursory epithelial cells and contribute to HNSCC malignancy [28]. 

Given the growing incidence of HNSCC, effective therapies need to be developed to reduce mobility and increase survival in these patients. Results of recent studies identify SphK1 as a potential modulator of carcinogenesis in head and neck cancer. Manipulating SphK1 levels may be an effective approach in treating HNSCC.

## 4. SphK1 Is a Major Player in HNSCC

SphK1 is the key enzyme, which balances the levels of bioactive sphingolipids ceramide, sphingosine and S1P. Several recent reports suggest that SphK1 plays an important role in head and neck carcinogenesis. For example, previous research has shown that SphK1 is overexpressed in HNSCC tumors [11,31], and esophageal [32] and thyroid carcinomas [33]. The role and mechanism of SphK1 in promoting head and neck malignancies have not been clearly delineated. The following four studies collectively show that SphK1 is positively associated with HNSCC, invasion, and reduced sensitivity to radiation treatment. Some studies offer potential mechanisms linking SphK1 to head and neck carcinogenesis, and they are outlined below. 

Using clinical samples, SphK1 overexpression was shown to be higher in SCC samples, and this was associated with depth of tumor invasion, metastasis, and clinical failure. For example, SphK1 expression was significantly higher in clinical human HNSCC samples compared to normal mucosa when measured with immunohistochemistry (IHC) and qPCR [31]. Two-hundred and forty-six HNSCC samples (mainly oral mucosa, pharynx, and larynx) with varying degrees of differentiation and different countries of origin (*i.e.*, North and South America, Africa, Southeast Asia) were compared to nonmalignant tissues collected from mouth floor, tongue, gingival, palate and other tissues (*i.e.*, breast, placenta, colon, lymph node, lung, skin, salivary gland). There were no correlations between SphK1 expression and grade, gender, primary tumor site or country of origin. In addition, in a cohort of 21 stage-III, male patients with identical treatment after surgery, SphK1 expression is negatively correlated with patient survival. In samples taken from these patients, SphK1 positive staining was associated with a 25-month survival period, whereas SphK1 negative staining was associated with about 80 months of survival post-surgery (Kaplan-Meir analysis) [31]. 

In another study, human esophageal carcinoma tissue samples were collected from 124 patients who were diagnosed with esophageal squamous cell carcinoma (ESCC), underwent surgery, did not receive neoadjuvant therapy prior to surgery and were followed for eight years post-surgery [32]. Tissue samples were stained for SphK1 expression and scored by intensity and percentage of staining on a scale of 1 to 3, which corresponded to <25%, 25%–50% or >50% staining, respectively. Kaplan-Meier analysis revealed that SphK1 expression (score ≥2) was significantly associated with clinical failure (three-year survival), while weak SphK1 staining (score <2) was associated with longer survival (eight years). Paired samples of normal and tumor tissues from ESCC patients showed SphK1 was indeed overexpressed in ESCC when measured both with Western blot and IHC. SphK1 expression also correlated with depth of tumor invasion, lymph node metastasis, and pathological state. At the end of the eight-year study, 117 patients had passed away. The extremely short survival rate in patients with SCC underscores the importance of understanding its physiology and pathology so that effective therapies can be developed and used. 

Furthermore, SphK1 was shown to be overexpressed in human HNSCC, where SphK1 exhibited strong staining in the cytoplasm, particularly the plasma membrane and surrounding macrophages and fibroblasts [11]. Normal mucosa had very little SphK1 staining [11]. Interestingly, SphK1 staining was observed in all stages of HNSCC, even in stage I, and there were no differences between SphK1 staining from stage I to IV. Based upon these data, the authors concluded that SphK1 is most likely involved in early stages of malignant transformation from normal mucosa to HNSCC [11].

To better understand the role of SphK1 in HNSCC progression, the authors used 4-nitroquinoline 1 oxide (4-NQO) to induce tongue carcinogenesis in SphK1 knockout (KO) and wild type (WT) mice [11]. SphK1 deficient mice exhibited reduced 4-NQO-induced tongue carcinogenesis. 72% of the SphK1 KO mice developed tumors while 96% of the WT mice developed tumors. SphK1 KO mice developed 1.2 tumors per mouse, while WT mice developed 2.1 tumors per mouse. In addition, tumors in SphK1 KO mice were about 5.5 times smaller than WT tumors (4.85 mm^3^ *versus* 27.02 mm^3^, respectively) [11]. SphK1 KO mice grew tumors with lower cell proliferation in HNSCC tumors compared to WT mice. BrdU labeling showed that 17.6% of cells from SphK1 KO mice entered the S-phase, while 23.4% of cells from WT mice entered S-phase. In addition, IHC showed that 50% of tumor cells from SphK1 KO mice stained positive for cleaved caspase-3 (indicative of apoptosis), *versus* 24% of tumor cells from WT mice [11]. 

The mechanism underlying the reduced tumorigenesis in SphK1 KO mice may have been due to reduced S1P, increased C16-ceramide levels, or reduced p-AKT. Extracellular S1P was significantly reduced in KO mice, which is not surprising since SphK1 converts sphingosine to S1P. Furthermore, C_16_-ceramide was reduced in KO mice [11]. This is consistent with previous research which documents up regulated C_16_-ceramide levels in HNSCC tumor tissues, concomitant with reduced C_18_-ceramide levels [12,34]. IHC staining for p-AKT (ser473) was also reduced in KO mice, suggesting that SphK1/S1P modulates downstream AKT signaling [11].

In addition to HSNCC, SphK1 is also associated with invasive ability of ESCC [32]. The investigators used a variety of ESCC (EC9706, KYSE30, KYSE150, KYSE510, KYSE2, NEC) lines to show that SphK1 was up regulated in KYSE2 and KYSE30 cell lines and this was associated with greater cell invasion (across transwell membranes). SphK1 overexpression in EC9706 cells resulted in greater invasive morphology and cell diameter compared to the parent cell and empty vector control cells. Interestingly, SphK1 up regulated proliferation (measured with cell viability assay) but did not influence apoptosis (measured with flow cytometry). 

The authors went on to show that immunodeficient mice subcutaneously injected with EC9706 cells overexpressing SphK1 developed tumors about twice as large and heavy compared to mice injected with parent or empty vector clones [32]. Mice injected with SphK1 overexpressing clones exhibited six-fold greater lung metastasis compared to parent cells. Microarray analysis showed that SphK1 expression correlates with genes downstream of the EGFR pathway (*i.e.*, amphiregulin, integrin_α__5_, epiregulin) [32]. In addition, SphK1 overexpressing cells had greater phosphorylation of EGFR, while cells transfected with siRNA against SphK1 showed reduced EGFR phosphorylation [32]. EGFR appears to be important in modulating invasiveness in ESCC. 

In a more recent effort to elucidate the role of SphK1 in HNSCC, Sinha and colleagues incorporated primary human HNSCC tumor tissues and murine HNSCC xenografts to show the apoptotic effect of silencing SphK1 on tumor growth [35]. Specifically, they showed elevated SphK1 expression in primary HNSCC tissues and lymph nodes, with highest expression with advanced stage of disease; and a positive correlation between SphK1 expression and recurrent human tumors. It is worthwhile to note that SphK1 was detected in lymph nodes, indicating a potential role of SphK1 in invasion and nodal metastasis. 

In the same study, BALB/C athymic mice were grafted with oral squamous cells of the tongue (SCC-15) transfected with either SphK1 siRNA or control GFP [35]. Mice receiving radiation and the SphK1 siRNA-graft had the greatest reduction in tumor volume (94%); mice receiving the SphK1 siRNA-graft had the next greatest reduction (67%); those mice receiving radiation (and the GFP-graft) had the smallest tumor volume reduction (17%). Lastly, they used IHC to show that *ex vivo* murine tumors treated with radiation and SphK1 siRNA-graft had greater pro-apoptotic caspase-3 expression and reduced Ki-67 staining (a marker of cell proliferation) compared to controls. The main finding was that silencing SphK1 reduced HNSCC tumor growth and sensitized tumors to radiation-induced death. Knockdown of SphK1 through *in vivo* delivery of SphK1-siRNA may be a therapeutic strategy to increase sensitivity of HNSCC tumors to radiation. While SphK1 was found to be a key player in tumor growth, downstream signaling remains to be elucidated [35]. 

Collectively, these studies indicate that SphK1 promotes cell proliferation, metastasis and invasion. In addition, increased SphK1 levels are associated with poor outcome, while lower SphK1 levels are associated with increased patient survival. 

## 5. SphK1 Influence in Head and Neck Cancer

**Figure 1:**
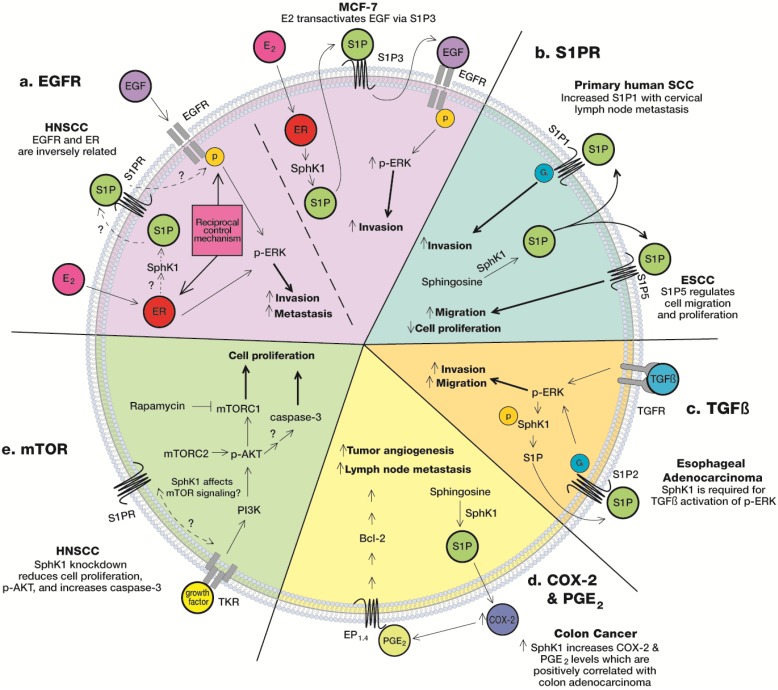
Suggested roles of SphK1 in HNSCC. EP: E-prostanoid receptor; TKR: tyrosine kinase receptor.

It is well-documented that SphK1 is a key player in tumor growth, but the mechanism underlying its influence on invasion and proliferation has not been fully elucidated. The effects of SphK1 on invasion are probably dependent upon S1P, as previous studies demonstrate interaction between S1PR and other cell surface receptors. For example, S1P has shown to interact with TGFβ receptors in esophageal cancer cells [39], EGFR in breast cancer cells [37], VEGFR in thyroid cells [49], and of course its own S1P receptors. Alternatively, SphK1 may affect the mTOR pathway [11] or SphK1 may stimulate COX-2/PGE_2_-proliferative pathways [40,41,42] to ultimately affect proliferation, invasion, metastasis and angiogenesis. These pathways have not been fully delineated and described in HNSCC. However, based upon HNSCC characteristics and its similarities to previous research in different cancer models, it is not farfetched to postulate that these relationships may also exist with HNSCC. The following section summarizes findings from previous studies that offer data, which suggest that these pathways are functional in HNSCC (Figure 1).

### 5.1. S1PR

The most obvious mechanism involves SphK1-mediated increases in S1P, and subsequent S1P binding to its receptors, S1PR. S1P binding to one of its five G-coupled protein receptors (GPCR) on the cell surface *via* autocrine and/or paracrine regulation can activate Rac, Ras-ERK, PI3K-AKT-Rac, phospholipase C (PLC), and Rho [6]. S1P binds to GPCR, where each receptor associates with one or more heterotrimeric G proteins, G_i_, G_q_ or G_12/13_. The signaling cascades stemming from S1P coupling to GPCR differ among the five receptors and the resultant action is dependent upon the G-protein coupled to the S1P receptor. For example, S1P_1_ (EDG1) associates specifically to G_i_ [50], while S1P_2_ (EDG5) and S1P_3_ (EDG3) couple to G_i_, G_q_ and G_13_ [50]; S1P_4_ (EDG6) couples to Gi [51,52] and G_12/13_ [53], and S1P_5_ (EDG8) associates with G_i_ and G_12_ [54]. G_i_ pathways are prolific pathways, while G_12/13_ work in opposition to inhibit proliferation and migration (previously reviewed in [55]).

While S1PR has been specifically studied in breast [56], gastric [57], thyroid [33,58], melanoma [59], and glioblastoma [60], research in HNSCC is sparse (as reviewed in [61]). To date, a literature search seeking peer-reviewed articles focusing on HNSCC and S1P-S1PR interaction results in a very limited number of studies.

Esophageal squamous carcinoma cells (Eca109) express S1P_1-3, 5_ and S1P_5_ was identified as an important receptor in regulating proliferation and migration [15]. Eca109 cells overexpressing S1P_5_ exhibited spindle cell morphology with elongated filopodia-like productions in the absence of S1P, which the authors identified as a marker of increased motility [15]. In addition, S1P_5_ overexpressing cells had higher migration through a transwell membrane compared to control cells in the presence of S1P [15]. These findings suggest S1P_5_ promotes migration.

In the same report, Hu *et al*. [15] also presented data suggesting that S1P_5_ is a tumor suppressor. Firstly, S1P_5_ was expressed at higher levels in normal mucosal epithelium compared to Eca109 cells. Secondly, S1P_5_ ovexpression in Eca109 cells reduced cell proliferation in the absence and presence of S1P. These two findings are consistent with that of Young *et al*. [62], who demonstrated that S1P_5_ inhibits cell proliferation in glioma cells. 

To add complexity to these findings, Hu *et al*. [15] also showed S1P_5_ overexpressing cells had increased migration through a transwell membrane in the absence of S1P, but reduced migration in the presence of S1P. This was interpreted as S1P_5_ are constitutively expressed in Eca109 in the absence of a stimulating ligand, but these cells may down-regulate S1P_5_ in a tumor microenvironment containing S1P to evade the inhibitory effect of S1P-S1P_5_ on migration. 

In glioma cells, Young and colleagues also showed dichotomy of S1PR regulation as S1P_1_, S1P_2_, and S1P_3_ enhanced S1P-stimulated cell proliferation, while S1P_1_ and S1P_3_, but not S1P_2_, enhanced invasion and migration [62]. It is possible that the same receptor can increase invasion and migration but differentially affects proliferation. In addition, Hu *et al*. [15] measured basal mRNA levels of S1P_1-5_, but the effect of the different receptors (*i.e.*, S1P_1-3_) on proliferation and migration was not examined. Therefore, it is unknown if S1PR expression levels were compensated by other S1PR in the absence and presence of ligands. It is difficult to fully and accurately interpret these findings. 

In a poster presentation, Ledgerwood *et al*. [38] illustrated significantly higher S1P_1_ expression levels (measured with IHC) in primary oral cavity SCC from patients with cervical lymph node metastasis (n = 30) compared to those without metastasis. In addition, primary tumors from patients without metastasis lacked S1P_1_ expression completely, while nine out the 30 nodal positive samples expressed S1P_1_. Twenty out of 30 metastatic lymph node samples had cancer cells with higher S1P_1_ expression compared to cells in primary tumors. While these data indicate that S1P_1_ and S1P_5_ are important in SCC, S1PR research in head and neck malignancies are sparse and continued research in this area is needed. 

### 5.2. Epidermal Growth Factor Receptor (EGFR)

The epidermal growth factor receptor (EGFR) is one of four cell-surface receptors in the family of ErbB tyrosine kinase receptors. EGFR are overexpressed in a variety of cancers and its upregulation is associated with poor prognosis and decreased survival. EGFR expression in patients is a strong prognostic indicator for overall survival and disease-free survival [63]. 

EGFR has been widely studied in head and neck cancer (previously reviewed in [64]). EGFR is overexpressed in 40%–90% of HNSCC [65,66,67] and is overexpressed in 43% of ESCC [68]. Truncated mutant EGFR variant III expression, in which exons 2–7 are deleted, was detected in 42% of HNSCC tumors, resulting in constitutive activation of EGFR, increased proliferation and tumor volume, and enhanced resistance to targeting wild-type EGFR [65]. In addition, microarray analysis of human HNSCC tumor samples showed that SphK1 expression correlates with genes downstream of the EGFR pathway in ESCC (*i.e.*, amphiregulin, integrin_α5_, epiregulin) [32]. 

Whether SphK1 is directly involved in activation of EGFR in head and neck cancer is unknown. Since HNSCC share common attributes with human breast cancer cells (*i.e.*, expression of EGFR, estrogen receptor (ER)), it is not unreasonable to draw from studies using human breast cancer cells (MCF-7). In these cells, estrogen (E_2_) was shown to transactivate EGFR through S1P_3_ [37]. Both E_2_ and S1P induced EGFR activation and downstream ERK1/2 activation, and both were blocked by pertussis toxin (PTX), a G_i_-specific inhibitor. This indicates that E_2_ and S1P act through a common signaling pathway to activate EGFR. 

Treatment with conditioned media collected from E_2_-treated cells resulted in an increase in EGFR tyrosine phosphorylation compared to cells treated with conditioned media derived from untreated cells [37]. Removing S1P from the conditioned media abolished EGFR activation induced by E_2_ indicating S1P is required for E_2_-induced transactivation of EGFR in MCF-7 cells [37]. 

E_2_ induced SphK1 phosphorylation in an ERK1/2 dependent manner in MCF-7 cells. Inhibition of ERK1/2 blocked E_2_-induced SphK1 phosphorylation and attenuated SphK1-mediated E_2_-induced EGFR activation. Blockade of EGFR activation resulted in inhibition of E_2_-induced ERK1/2 activity. Thus, the authors suggest that ERK1/2 can exist either upstream or downstream of SphK1 signaling, as it “has a dual role in initiation and amplification of a positive-feedback signaling loop across E_2_, SphK1 and EGFR in breast cancer cells [37].” The authors concluded that S1P and its receptors are critical in the E_2_-stimulated activation of EGFR, where SphK1 couples or mediates signaling between E_2_, S1P and EGF in a “criss-cross” manner [37]. Thus, S1PR is an integral part of this pathway, as perturbation of its activity affects downstream EGFR signaling. 

While the SphK1/S1P axis has not been shown to transactivate EGFR in HNSCC, cross-talk between ER and EGFR has been shown in various HNSCC cell lines cultured from male and female donors [36]. Egloff and colleagues used an ERE luciferase construct (gene reporter assay) to show that E_2_ stimulation activated ER transcription, proving endogenous ER are functional in HNSCC. The level of ER transcription was inversely related to EGFR protein expression, where cells with the lowest EGFR protein expression exhibited greatest ER transcription. This was recognized as a reciprocal control mechanism. E_2_ normally stimulates p-ERK, but the addition of EGF ligand neutralizing antibodies (against amphiregulin, heparin-binding-EGF and TGFα) and EGFR neutralizing antibody (M225) abrogated the E_2_-induced phosphorylation of ERK1/2. Furthermore, inhibition of matrix metalloprotease (MMP) with marimastat abrogated E_2_-induced phosphorylation of ERK1/2. These observations indicate functional interaction between ER and EGFR in HSNCC, where E_2_-induced stimulation is dependent upon both EGFR and MMP [36]. This is reminiscent of data presented by Sukocheva *et al*. [37], who showed MMP inhibition blocked E_2_ and S1P-induced EGFR activation in MCF-7 breast cancer cells.

Egloff *et al*. [36] used carcinoma of the epiglottis (PCI-37A) to show treatment of E_2_ or EGFR alone increased invasion through a matrigel matrix system (~4×), and combination treatment of E_2_ plus EGFR maximally induced cell invasion (~6×). In addition, individual inhibition of E_2_ and EGFR (with gefitinib and fulvestrant, respectively) inhibited invasion by 52- and 47-fold, respectively, while combined inhibition resulted in even greater inhibition (74-fold reduction in invasion). Along the same lines, patients with high tumor nuclear ERα_(nucl)_ and high EGFR have significantly reduced progressive free survival (hazard ratio: 4.09) compared to patients with low ERα_nucl_ and low EGFR levels, as estimated with Kaplan-Meier analyses. This hazard ratio is greater than that of patients with high ERα_nucl_, ERβ_nucl_, or EGF staining alone (hazard ratio: 2.27, 1.16, and 1.95, respectively) [36]. Although it is likely that there is a redundancy in these pathways, the authors conclude that ER and EGFR interact to increase invasion and proliferation. It is worthwhile to note that although no sex-based differences in ER expression levels were found in HNSCC cell lines or tumors, high ERα_nucl_ levels were associated with reduced progression free survival in women, a trend not seen in males [36]. This suggests the possibility that ER activity may be a factor in determining tobacco-related susceptibility between sexes. Based upon these findings, and the similarity to data presented in MCF-7 cells [37], it is not unlikely that SphK1/S1P is involved in mediating the interaction between ER and EGFR. 

It is unclear if SphK1/S1P is involved in EGFR/ER signaling in HNSCC. Further research is needed to identify if SphK1/S1P is involved with these cell surface receptors and elucidate the relationship between them. 

### 5.3. mTOR

The mammalian target of rapamycin (mTOR) is a serine/threonine kinase and a member of the phosphatidylinositol 3 (PI-3)-kinase-like kinases. mTOR is involved in various signal transduction pathways controlling cell growth, proliferation, and survival. As seen in other types of cancers, AKT/mTOR are dysregulated in 90%–100% of HNSCC [69] and this is associated with poor prognosis [70,71]. mTOR dysregulation is thought to be an early event because dysregulation is detected in dysplastic lesions [72]. Based upon the findings that p-AKT (ser473) was reduced in tongue tumors induced by 4-NQO in SphK1 KO mice [11] and mTOR inhibitor, rapamycin, significantly reduced malignant conversion of precancerous lesions and promoted regression of 4-NQO induced oral carcinogenesis [72], Shirai *et al*. [11] postulates that SphK1/S1P modulates downstream AKT signaling and plays a role in dysregulating mTOR signaling. No additional work was done in this area to test this hypothesis. Research determining how SphK1 and AKT are involved in head and neck carcinogenesis is required.

In a different study, two highly invasive human HNSCC cell lines (UMSCC2 & UMSCC17B) were orthotopically injected into the tongue of SCID/NOD mice, and these cells grew as highly aggressive tumors, invading the muscle & surrounding tissues. Treatment with mTOR inhibitors (rapamycin and rapamycin analog RAD001) reduced growth of primary orthotopic HNSCC tumors, prevented the metastatic spread of primary HNSCC lesions to cervical lymph nodes, reduced intratumoral lymphangiogenesis, and increased survival in mice [2]. Diabetes drug, metformin has also been shown to inhibit mTORC1 activity *via* an AMPK-independent action, retarding tumor size and the number of 4-NQO-induced oral tumoral lesions [73]. Clearly, mTOR is an important signaling pathway in HNSCC. However, aside from its regulation of p-AKT (ser 473) [11], SphK1 has not been directly linked to any other component of mTOR signaling in HNSCC. Additional work in this area is needed.

### 5.4. COX-2 & PGE_2_

Cyclooxygenase(COX)-2 is an inducible enzyme which converts arachidonic acid (AA) to prostaglandins including prostaglandin E_2_ (PGE_2_), which induces inflammation and is involved in various cancers. COX-2 is overexpressed in HNSCC and oral premalignant lesions [74]. COX-2 is not constitutively expressed, but it can be induced by benzo[a]pyrene (a carcinogen found in diesel engine exhaust fumes and cigarette smoke) [75], and other factors involved with inflammation and tumorigenesis [76]. COX-2 catalyzes the synthesis of PGE_2_ [74], where increased PGE_2_ production promotes cell survival *via* upregulation of pro-survival Bcl-2 expression in HNSCC [45]. 

COX-2 and PGE_2_ are associated with tumor angiogenesis and lymph node metastasis in HNSCC [40]. COX-2 and PGE_2_ are higher in human HNSCC biopsies *versus* normal mucosa, with patients with lymph node metastasis having higher COX-2 protein expression and PGE_2_ levels compared to patients without metastasis (as measured with IHC) [40]. In another study using HNSCC patient biopsies, COX-2 (measured with RT-PCR) and cytosolic PGE-synthase, an enzyme that generates PGE_2_, was significantly elevated 4- and 2.5-fold, respectively, compared to normal mucosa [41]. In a separate study, COX-2 mRNA was ~150-fold higher in HNSCC when compared to normal mucosa, and COX-2 protein was elevated in HNSCC, but not detected in normal mucosa [42]. These observations were also consistent with another study which showed oral squamous carcinoma tissues had greater COX-2 mRNA expression when compared to normal tissues [44].

The COX-2/PGE_2_ pathway is also stimulated in animal models, where COX-2 protein was up-regulated six-fold in 4-NQO-induced SCC of tongue epithelia as measured with Western blot [43]. A selective COX-2 inhibitor, nimesulide, was effective in reducing the incidence and multiplicity of SCC [43,77]. In addition, COX-2 was up-regulated in response to smokeless tobacco extract in hamster cheek pouch epithelial cells (HCPC-1). In this study, moist snuff was prepared in solution and suffused over hamster cheek pouch for 20 min and then animals were euthanized. PGE_2_ was used as an indirect measure of COX-2. PGE_2_ reflected dose- and time-dependent increases in response to smokeless tobacco in HCPC-1 *ex vivo*. Furthermore, authors show that anti-inflammatory protein annexin I was cleaved in response to smokeless tobacco exposure and the loss of annexin I may account for overexpression of COX-2 [44].

While these aforementioned studies do not directly indicate that SphK1 mediates COX-2 and PGE_2_ expression in HNSCC, this role has been described in colon carcinogenesis. Azoxymethane (AOM)-induced adenocarcimonas were positively correlated with strong SphK1 staining [78]. Kawamori *et al*. [48] also showed siRNA downregulation of SphK1 decreased COX-2 expression/PGE_2_ production, and S1P stimulated COX-2/PGE_2_ production in human colon cancer cells (HT-29). Since COX-2 and PGE_-2_ are elevated in HNSCC [40,42,79], it is not unreasonable to theorize that SphK1 could regulate the COX-2/PGE-_2_ pathway in HNSCC. 

COX-2 may be an integral player in HNSCC pathology. Still, COX-2 therapies would most likely need to be used in combination with other treatments. However, clinical trials have been unable to show efficacy of celecoxib (a specific COX-2 inhibitor) [80] and Ketorolac (COX-1 and -2 inhibitor) [81] in inhibiting oral leukoplakia and oral premaligant lesions, respectively. Non-steroidal anti-inflammatory drugs (NSAIDs), a known PGE_2_ inhibitor, had no effect on HNSCC recurrence or survival when compared to non-users in a retrospective case-control study [82]. Thus, COX-2 therapies may be most effective when used with other therapies. For example, concurrent treatment of celecoxib and erlotinib (EGFR inhibitor) enhanced radiosensitivty in a phase I clinical study using HNSCC patients [83]. In fact, this report shows that this treatment was effective in treating a massive HNSCC tumor in the oropharayngeal wall and cervical lymph node of a patient who failed prior chemoradiation, had a total laryngectomy and neck dissection. The patient had a complete response to the treatment, indicating that EGFR/COX-2 treatment is a clinically feasible approach [83]. 

In conclusion, we know that elevated levels of SphK1 increase S1P levels to regulate COX-2/PGE_2_-mediated colon carcinogenesis [48,78]. We also know that COX-2 is overexpressed in head and neck cancer [42,84,85]. However, how SphK1, COX-2 and PGE_2_ work together to control HNSCC is not clear. 

### 5.5. TGFβ

TGFβ is known to play a dual role in epithelial cancer development as it acts as a growth inhibitor with tumor-promoting activities. It can induce reversible growth arrest in G1, and on the other hand, promote epithelial mesenchymal transition, migration, invasion, and carcinogenesis [86,87]. The TGFβ growth arrest cascade consists of activation of its receptors (TGFβ receptors I and II), Smad2 and Smad3 phosphorylation, and Smad nuclear entry/action [88]. TGFβ also activates tumor promoting pathways, namely ERK1/2, p38 MAPK (mitogen-activated protein kinase), JNK, and PI3K (phosphatidylinositol 3-kinase) [89].

Interaction between S1P and TGFβ is well-established in a variety of cell lines [90,91,92,93,94]. S1P stimulates phosphorylation of Smad2 and Smad3 in keratinocytes [92], S1P activates TGFβR and Smad in rat mesangial cells [93], and S1P_3_ is involved in Smad3 signaling in myofibroblast differentiation [94]. 

In head and neck cancer, Miller *et al*. [39] demonstrated that S1P interacts with TGFβ to affect invasion and migration of esophageal cancer cells in human esophageal adenocarcinoma (OE33). In this study, invasion was defined as a measure of the number of cells that degraded and moved through the matrigel, whereas migration was a measure of the number of cell that travelled into the lower chamber of the transwell model. In OE33 cells, they showed (1) ERK1/2 activation, migration and invasion are SphK1- and G_i_-dependent; (2) knockdown of SphK1 with siRNA reduced migration by 50%, while knockdown of SphK2 reduced migration by about 25%; (3) invasion was reduced with siRNA knockdown of SphK1, but not SphK2; (4) TGFβ stimulation caused phosphorylation of SphK1 (Ser225) in a time-dependent manner concomitant with increased S1P in cell lysates, and (5) S1P_2_ knockdown blocked TGFβ-induced ERK1/2 phosphorylation, migration and invasion.

TGFβ induced SphK1 phosphorylation at residue Ser225 and subsequently increased S1P concentration 15 min post-stimulation. Surprisingly, SphK1 protein levels were not affected despite the increased SphK1 activity. PTX (a specific G_i_ inhibitor) and SphK1 inhibitor DMS (N,N-dimethylsphingosine) reduced S1P- and TGFβ-induced ERK1/2 activation, migration and invasion, indicating both S1P and TGFβ activation of ERK1/2 are G_i_-dependent. Together these observations suggest that TGFβ modulates invasion *via* an SphK1/S1P/S1PR-dependent mechanism. Notably, SphK1, and SphK2 activity levels were independent of protein expression, as SphK activity increased for both kinases but mRNA remained unchanged [39]. 

In OE33 cells, S1P*_2_* and S1P*_5_* are present in high levels, S1P_1_ and S1P_3_ are present in low levels, and S1P_4_ is not detectable [39]. To pinpoint the involved S1P receptor, JTE013, a S1P_2_ antagonist and VPC23019 (S1P_1_/S1P_3_ antagonist) were used. S1P_2_ antagonist (JTE013), but not the S1P_1_/S1P_3_ antagonist (VPC23019) blocked TGFβ- and S1P- induced invasion and migration. S1P_2_ downregulation significantly inhibited S1P-and TGFβ-induced ERK1/2 activation, cell migration and invasion, indicating S1P_2_ is critical in the TGFβ pathway. Downregulation of S1P_2_ did not affect the other S1P receptors. S1P_2_ is thought to inhibit migration largely due to the G_12/13_-dependent inhibition of Rho and Rac [62,95,96]. However, since inhibiting S1P_2_ attenuated invasion and migration, the authors believe that S1P_2_ may be coupled primarily to G_i_ and not G_13_. TGFβ-induced activation of ERK1/2 occurred within 15 min and since Smad activation can take several hours to activate [97], the authors deduced the TGFβ activation of ERK1/2 was Smad-independent [39]. TGFβ-S1P interaction is a plausible pathway in which SphK1 mediates cell invasion and migration in SCC. However, this conclusion is supported mainly by only one study and thus, additional work in this area is required. 

While the underlying mechanism of SphK1 in SCC is not definitive, the influence of SphK1 is probably due to a combination of pathways as outlined above (*i.e.*, EGFR, S1PR, mTOR, COX-2/PGE2, TGFβ). It is difficult to affirmatively state which pathway is the predominant factor because these studies have used different models, making comparison difficult. In addition, there are only a few studies focused on each of these pathways mentioned above. Without many studies to review, it is difficult to ascertain the mechanism of SphK1 in HNSCC. Systematic methodologies among future studies and more research on a whole are necessary to draw definitive conclusions about Sphk1’s role in HNSCC.

## 6. SphK1/S1P Pathway and Thyroid Cancer: Mechanism of Action

Thyroid cancer is also a type of head and neck cancer, but originates from follicular epithelial cells, and therefore is pathologically different from HNSCC. However, research using this model has provided useful information, and should not be overlooked. Papillary and follicular carcinoma account for the most common forms of the well-differentiated carcinoma [98], and follicular and anaplastic carcinoma account for the majority of poorly-differentiated carcinomas [99]. This next section reviews studies that describe SphK1’s role in thyroid cancer. 

As seen in HNSCC, SphK1 is overexpressed in human thyroid cancer and expression levels correlate with the degree of malignancy. More specifically, 69% (29 out of 42) of thyroid cancer samples analyzed elicited SphK1 overexpressed as measured with IHC [33]. In addition, high SphK1 expression was observed in all 10 anaplastic tumor cancer (ATC) specimens examined. ATC has poor prognosis due to its aggressiveness (high mitotic rate and lymphovascular invasion) and resistance to treatment. In contrast, not all papillary thyroid cancer and follicular thyroid cancer samples examined stained strongly for SphK1. 59% (13 out of 22) and 60% (6 out of 10) of papillary thyroid cancer and follicular thyroid cancer, respectively, exhibited high SphK1 staining. These data indicate that SphK1 is associated with the degree of malignancy in thyroid cancer. 

shRNA targeted knockdown of SphK1 resulted in reduced proliferation, and the number of floatage-independent colonies in thyroid cancer cell lines WRO (follicular), FRO (anaplastic), and S579 (poorly differentiated cancer with feature of papillary cancer) [100]. shRNA targeting SphK1 in thyroid cancer cells resulted in a cascade potentially increasing cell motility [100]. The specific cascade consisted of dephosphorylation (and inactivation) of Akt (ser473/thr308), dephosphorylation (and activation) of GSK-3β, decreased β-catenin protein in nuclear fractions (indicative of inactivation), and increased phosphorylation of β-catenin at Ser 33/Ser37/Thr41 (leading to degradation and inactivation of β-catenin) [100]. β-catenin-T cell factor/lymphoid enhancing factor (TCF/LEF) is not only a component in Wnt signaling, but it also floats actin cytoskeleton and plays an integral role in cell-to-cell adhesions. These observations are consistent with other reports of dysregulation of β-catenin in colon cancer, endometrial cancer, ovarian cancer, hepatocellular carcinoma, medulloblastoma, and melanoma (reviewed in [101]).

A series of studies confirm that SphK1 enhances cell migration in human thyroid follicular carcinoma cells (ML-1) [102]. Consistent with an enhanced migratory phenotype, SphK1 overexpression reduced adhesion of ML-1 cells to human collagen IV coated plates when compared to control cells [58]. In fact, SphK1 and S1P induced migration of ML-1 cells *via* activation of S1P_1_ and S1P_3_, G_i_ proteins and the PI3K-Akt pathway [102]. Overexpression of SphK1 in ML-1 cells enhanced S1P secretion and subsequent S1P action through autocrine activation. This increased migratory flux could be reversed by reducing S1P secretion with SphK inhibition and an ATP-binding cassette transporter which blocks S1P release from the cell. Together, these findings indicate that SphK1 and S1P regulate migration in an autocrine fashion in ML-1 cells [102]. 

PKC and ERK1/2 are also necessary for S1P-induced migration in ML-1 cells [102]. Inhibition of PKC-α and PKC-β1 with PKC inhibitor Go6971 resulted in inhibition of S1P-induced phosphorylation of ERK 1/2 [102]. In addition, PKC-α siRNA attenuated serum-induced migration in cells overexpressing SphK1, suggesting that PKC is necessary for S1P-induced migration. Along the same lines, inhibition of ERK1/2 by U0126 attenuated migration in SphK1 overexpressing cells, indicating that ERK1/2 is also essential to S1P-induced migration. The authors concluded that PKC and ERK1/2 may be the main mediators in serum-induced migration. They summarized their findings by stating that S1P acts on its cell surface receptor to activate PKC-α, which can stimulate both ERK1/2 and SphK1 (to increase S1P production and “inside-out” signaling) to stimulate S1PR in a repetitive autocrine cycle, resulting in sustained migration [102]. 

In a different study, Balthasar *et al*. [49] demonstrated that S1P works with VEGFR-2 to regulate migratory responses in ML-1 and FRO cells (anaplastic thyroid cancer cell). Treatment of ML-1 and FRO cells with S1P stimulated secretion of VEGF-A, while blocking S1P receptors (S1P_1_, S1P_2_, S1P_3_) and reduced VEGF-A secretion. In addition, they showed that S1P phosphorylates VEGFR-2 in ML-1 cells, indicating that S1P is capable of regulating VEGF-A. In addition, rapid downregulation of S1P_1_ protein and mRNA expression occurred with inhibition of VEGFR-2 in ML-1 cells, indicating that VEGF also regulates SphK1. Moreover, inhibition of VEGFR-2: (1) attenuated migration through a transwell membrane in both ML-1 and FRO cells; (2) suppressed S1P_1_ protein and mRNA expression; (3) suppressed S1P-induced migration; and (4) reduced S1P induced phosphorylation of AKT (ser473). 

While it appears that S1P and VEGFR regulation works in unison, the interaction between S1P and VEGFR is not clear. Inhibition with VEGFR-2-inhibitor 1 (which inhibits receptor tyrosine kinase activity), but not neutralizing antibodies or inhibitor CBO-P11n (which interferes with VEGF-A/VEGFR interaction) reduced cell ML-1 cell proliferation independently of S1P stimulation. In addition, inhibition of VEGFR-2 increased S1P_3_ mRNA but not S1P_3_ protein levels. Nevertheless, on a whole, data from this study suggest that VEGFR and S1P act together to increase proliferation, invasion and migration [49]. These findings are consistent with previous studies, which showed that SphK1 overexpression increases invasion in esophageal SCC [32] and ML-1 cells [102]. These findings are also in line with data showing SphK1 overexpression in patient HNSCC samples correlate with reduced survival time [103]. 

In a follow-up study, Bergelin *et al*. [58] showed that S1P_1_ and VEGFR-2 co-localize at plasma membrane domains and regulate PKC-α and ERK1/2 signaling in ML-1 cells. In fact, VEGFR-2, ERK1/2, and PKC- α were immunoprecipitated with each S1PR in ML-1 cells, indicating physical interaction among these components. In addition, PKC-α inhibition abrogated S1P_1_ and VEGF-A induced ERK1/2 phosphorylation, indicating functional interaction among these players. 

Bergelin *et al*. [58] also showed that VEGFR-2 inhibition attenuated ERK1/2 phosphorylation when stimulated with S1P_1_ agonist (SEW-2871) and S1P [58]. S1P_1_ inhibition attenuated ERK1/2 phosphorylation when stimulated with VEGF-A. Down regulation of SphK1 with siRNA inhibited VEGF-A-induced ERK/12 phosphorylation, indicating S1P is required for VEGF-induced ERK1/2 phosphorylation. Together this suggests that while S1P_1_/VEGFR-2 crosstalk is bidirectional (S1P_1_ and VEGFR-2 regulate each other), S1P_1_ is a key factor in regulating ERK1/2. In fact, SphK1 knockdown in ML-1 cells overexpressing SphK1 reduced basal levels of phosphorylated ERK1/2 further suggesting S1P is the primary regulator of ERK. Specifically, S1P_1-3_ regulates phosphorylation of ERK1/2 through G_i/o_ proteins. This was demonstrated as S1P_1_/S1P_3_ antagonist (VPC23019), S1P_2_ antagonist (JTE013) and PTX (G_i_-specific inhibitor) treatment inhibited ERK1/2 phosphorylation. In addition, siRNA targeted S1P_1_ downregulation blocked S1P- and VEGF-A-induced directional motility toward collagen (haptotaxis), further showing S1P-S1PR activation is critical in this pathway. The authors concluded that S1P_1_ and VEGF-A independently stimulate ERK1/2 phosphorylation through PKC-α. Taken together, these studies demonstrate that SphK1 is overexpressed in thyroid carcinoma, and SphK1/S1P is critical in regulating migration [33,49,58,100,102].

Research in thyroid cancer has identified that ERK1/2 and PKC is responsible for modulating SphK1/S1P-stimulated migration. The use of similar models and continuity in these studies elucidate that SphK1 regulates migration in thyroid cancer *via* a S1P-PKCα-ERK1/2 pathway. These studies provide an excellent model for SphK1-HNSCC research. 

## 7. Other Sphingolipid Mediators and HNSCC

**Figure 2:**
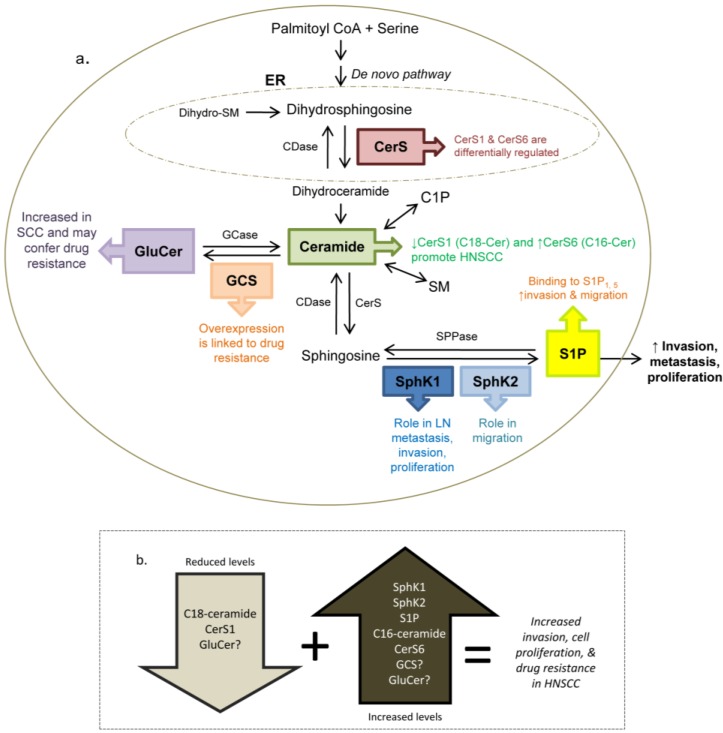
Overview of sphingolipid enzymes and metabolites and their influence on HNSCC. (**a**) **S**phK1 is positively associated with increased lymph node (LN) metastasis [32], proliferation [11] and resistance to radiation-induced cell death. (**b**) Perturbation of specific sphingolipid players affects invasion, proliferation and drug resistance in HNSCC. Abbreviations: SPPase (sphingosine phosphate phosphatase), GCS (glucosyl ceramidase), GCase (glucosyl ceramidase), CDase (ceramidase), CerS (ceramide synthase), C1P (ceramide 1-phosphate), SM (sphingomyelin), ER (endoplasmic reticulum).

Sphingolipids do not exist in isolation and perturbation of one metabolite or enzyme affects surrounding metabolites. Therefore, it is important to review research involving other sphingolipid related players in HSNCC, namely SphK2, ceramide, and glucosylceramide. These mediators have been shown to be of importance in HNSCC, and they are outlined in Figure 2. 

### 7.1. Sphingosine Kinase 2

The function of Sphk2 in head and neck cancer has not been widely examined. One of the few studies focusing on head and neck cancer reported that SphK2 knockdown in human esophageal adenocarcinoma (OE33) reduced migration (but not invasion) *in vitro* [39]. In addition, recent evidence shows SphK2 may play a critical role in regulating cell proliferation and apoptosis in other types of cancer, *i.e.*, mammary adenocarcinoma, hepatocellular carcinoma, leukemia [109,110]. Use of specific SphK2 inhibitor ABC294640, [3-(4-chlorophenyl)-adamantane-1-carboxylic acid (pyridin-4-ylmethyl)amide], results in cell death and inhibition of tumor growth in kidney, prostate and breast tumor cell lines [111,112,113,114,115]. Another specific SphK2 inhibitor, K145 (3-(2-amino-ethyl)-5-[3-(4-butoxyl-phenyl)-propylidene]-thiazolidine-2,4-dione)), reduced S1P levels in leukemia cells and demonstrated growth inhibitory and apoptotic effects in murine models [116]. Surprisingly, administration of SLR080811 ((S)-2-[3-(4-octylphenyl)-1,2,4-oxadiazol-5-yl] pyrrolidine-1-carboximidamide), a cationic amphiphilic small molecule and specific SphK2 inhibitor, resulted in a rapid increase in blood S1P [117]. This response is in contrast to SphK1 inhibitor administration, which resulted in down-regulated S1P levels. In fact, this response reflects a true physiological S1P response to SphK knockdown. Measurement of circulating S1P levels revealed that SphK1 KO mice had reduced S1P levels, while SphK2 KO mice had increased S1P levels [117]. The use of specific SphK2 inhibitors demonstrates that SphK2 is an important and influential modulator of carcinogenesis. Thus, while SphK1 has been the main focus in HNSCC, additional research is needed to determine the extent of SphK2 influence. 

### 7.2. Ceramide

SphK1 is differentially regulated in HNSCC, and it is possible that perturbations in SphK1 activity may affect other sphingolipids, which could exacerbate or reduce tumor progression. For example, SphK1 siRNA knockdown induces significant (1.5-fold) increases in ceramide levels in whole cells and mitochondrial fractions of MCF-7 cells [118]. In fact, SphK1 modulates total ceramide levels, ceramide subspecies, and subcellular distributions of growth-inhibiting ceramide [118]. Thus, we cannot overlook the possibility that other sphingolipids, such as ceramide, are the basis of tumor regulation in HNSCC.

The role of ceramide in directing tumor progression in cancer and specifically in HNSCC has been reviewed previously [3,34,119,120]. However, since ceramide is highly relevant and exerts considerable control over HNSCC tumor progression, a brief summary will be presented. Ceramide species differentially regulate tumor growth and invasion [12,34], namely ceramide synthase-1 (CerS1, also known as longevity assurance gene 1, LASS1) and ceramide synthase-6 (CerS6 or LASS6). CerS1 regulates C18-ceramide synthesis, while CerS6 affects C16-ceramide production [14]. 

C18:0 ceramide was the only ceramide selectively down-regulated in 19 out of 32 patient HNSCC tumor samples as measured with LC/MS [12]. This was also exemplified in another cohort of 45 HNSCC patients, where C18-ceramide was the only ceramide species down-regulated in tumors. In addition, the extent of C18-ceramide (both C18 and C18:1) deficiency was associated with higher incidence of lymphovascular invasion, pathologic nodal status, and higher stages of primary HNSCC tumors [34]. In contrast to the reduced C18-ceramides, C24 and C24:1 ceramides were significantly elevated in tumor tissues [34]. This exemplifies the differential regulation of ceramide subspecies in HNSCC tumors.

Another study showed the impact of CerS1 (LASS1) perturbation on HNSCC growth. Longevity assurance gene 1 (mLAG1) is a mouse homologue of mammalian upstream of growth and differentiation factor-1 (UOG1), or LASS1 and it regulates C18-ceramide synthesis with a high degree of specificity [121]. Koybasi *et al*. [12] overexpressed mUOG1, which resulted in upregulation of C18-ceramide. This substantially inhibited cell growth of UM-SCC-22A, SCC of the hypopharynx *in vitro* by 70%–80% through involvement of telomerase and mitochondrial dysfunction [12]. 

Another study showed that overexpression of CerS1 improved growth inhibitory effects of combination treatment of chemotherapy drugs gemcitabine and doxorubicin (GEM/DOX) in HNSCC cell line UM-SCC-22A [104]. These authors illustrated 1) combination treatment of GEM/DOX increased CerS1 mRNA by 30% in UM-SCC-22A cells, 2) CerS1 overexpression and subsequent C18-ceramide upregulation enhanced GEM/DOX -induced cell death through caspase-3 activation, and 3) GEM/DOX treatment in SCID mice with UM-SCC-22A xenografts inhibited tumor growth, and these chemically treated tumors showed a seven-fold increase in C18-cermide concomitant with decreased C16-ceramide levels [104]. Thus, increased C18-ceramide and reduced C16-ceramide levels are associated with retardation of tumor growth. 

More recently, a phase II clinical study which employed two cycles of combination GEM (1000 mg/m^2^)/DOX (25 mg/m^2^) treatment in patients with recurrent HNSCC identified serum C-18 ceramide as a viable biomarker of chemotherapy response [105]. Out of the 17 patients treated with GEM/DOX, one exhibited complete response, three had partial response, eight had stable disease, and five patients experienced progressive disease. Patients who responded to the treatment had significantly higher serum C18-ceramide levels compared to patients with progressive disease. The most common toxicity was neutropenia, affecting nine out of 18 patients. Remarkably, there were no other major non-hematologic toxicities. These results indicate a possible and effective treatment for patients with metastatic and/or reoccurring HNSCC [105]. It should also be noted that the role of CerS1/C18 ceramide and CerS6/C16-ceramide (and C24-ceramide) is suspected to be specific to squamous cells because the fluctuations of these enzymes and ceramide species are not consistent with those seen in non-squamous head and neck tumors [104]. Koybasi’s groups showed that non-squamous head and neck tumors exhibit lower levels of all three ceramide subspecies: C16-, C18-, and C-24 ceramide [12].

Photodynamic therapy (PDT) is a clinically approved procedure that selectively applies cytotoxicity toward malignant cells. It requires three components: a photosensitizer, light, and oxygen. This technique applies a light-absorbing photosensitizer, followed by irradiation at the specific wavelength that corresponds to the absorbance band of the sensitizer. The addition of oxygen initiates a sequence of events, involving reactive oxygen species, which leads to apoptosis (reviewed in [122]). Whether this treatment will be effective in HNSCC is questionable because this therapy is ineffective against metastatic lesions, which is very common in HNSCC. 

Nevertheless, PDT is of interest because PDT results in ceramide accumulation as cells undergo apoptosis [123]. In the absence of serine palmitoyltransferase, PDT inhibits sphingomyelin synthase and glucosylceramide synthase, causing ceramide accumulation. In a series of articles, Separovic *et al*. [124,125] demonstrated that reduction of CerS1 led to apoptotic resistance after PDT [124], while CerS6 knockdown reduced PDT-induced apoptosis in UM-SCC-22A *in vitro* [125]. This is consistent with previous data illustrating CerS1/C18-ceramide overexpression inhibits cell growth [12], and increased CerS6/C16-ceramide’s prosurvival role against ER-stress induced apoptosis in HNSCC [126]. It is well-documented that C18-ceramide/CerS1 are reduced and C16-ceramide/CerS6 is increased in HNSCC and perturbations in these enzymes and ceramide modulates response to chemo- and photodynamic-therapy.

The effects of ceramide in HNSCC have been well-documented. Collectively, these studies have demonstrated that ceramide is influential in affecting tumor growth. GEM/DOX treatments resulting in increased C18- ceramide and decreased C16-ceramide levels in clinical trials show the importance of ceramide in head and neck cancer.

### 7.3. Glucosylceramide

Glucosylceramide (GluCer) is produced through conversion of ceramide by enzyme glucosylceramide synthase (GCS). GSC is over expressed in a breast, skin, brain, ovary, and colon cancer cells (reviewed in [119]). Inhibition of ceramide formation by increasing GCS results in development of drug resistance in breast cancer cells [127], while inhibition of GCS reverses drug resistance through endogenous ceramide accumulation as seen in myeloid leukemia cells [128]. GCS overexpression is linked to drug resistance [106,107] and inhibition of GCS restores sensitivity to drug therapy [106,129] by controlling apoptotic ceramide levels. 

GCS is thought to work with ABC transporter, P-glycoprotein (P-gp) in conferring drug resistance, where either inhibition of GCS or P-gp reduces glucosylceramide translocation [45,119,130] for the synthesis of neutral glycosphingolipids [131]. However, inhibition of GCS has limited therapy potential [18]. Exogenous ceramide was effective in stimulating apoptosis, even with conversion by GCS. In addition, while GCS can convert *de novo* ceramide, GCS is not effective at catalyzing the conversion of ceramide derived from sphingomyelin [18]. Therefore, employing GCS inhibitors may not be as effective as modulating ceramide levels directly.

The limited potential of GCS inhibitors on HNSCC is probably the reason why research in this area is limited. However, one study reported that glycosphingolipid expression was significantly greater in 33 human samples of upper airway and digestive tract SCC compared to normal mucosa [108]. Specifically, ganglioside GM3, globoside, ceramide disaccharide (CDH), and ceramide trisaccharide (CTH) were two to three times higher in SCC *versus* normal tissue. 

Still, there is a possibility that elevated levels of GluCer are responsible for drug-resistance observed as suggested by the following studies. Breast cancer and melanoma patients responsive to chemotherapy had low GluCer levels, while GluCer levels were elevated in those who failed chemotherapy [132]. In addition, accumulation of GluCer is associated with blood lymphoma [133] and epidermal mitosis and proliferation [134]. β-glucocerebrosidase (GBA) is the enzyme that cleaves the beta-glucosidic linkage of glucocerebroside (glucosylceramide) and works in opposition to GCS to generate ceramide. Topical application of conduritol B epoxide (CBE), a specific inhibitor of GBA, and intracutaneous injections of GluCer stimulated epidermal proliferation. Combination treatment of CBE plus GluCer resulted in an additive increase in DNA synthesis [134], and epidermal hyperplasia [135]. These observations suggest GluCer accumulation leads to cell proliferation and tumor growth. 

In contrast, rice bran GluCer-feeding reduced tumor volume by one-half in mice. Subcutaneous xenografts of SCCKN cells (a well-differentiated recurrent SCC of the mouth floor) were implanted in NOD/SCID mice and allowed to proliferate for two weeks [136]. Tumors removed from mice fed dietary GluCer exhibited greater apoptosis as measured with TUNEL and greater cleaved (active) caspase-3 compared to control tumors. The authors reasoned that the dietary GluCer administered was C18:0-glyucosylceramide and may be coverted to C18:0-ceramide to inhibit proliferation. This is in accordance with another report which showed oral feeding of monoglucosylceramide, 1-O-beta-glucosyl-N-2'-hydroxyarachidoyl-4,8-sphingadienine, inhibited aberrant crypt formation induced by 1,2- dimethylhydrazine (DMH) during colon carcinogenesis [137].

These contradictory findings are not entirely surprising as the roles of GluCer (and GCS) are not clearly delineated in multi-drug resistance and the retardation of cell growth. Ultimately, its effects are probably tumor and cell specific [3]. Despite the attention GluCer has received in regulating drug resistance, GBA has received little to no attention in HNSCC. While GCS itself may have limited potential in treating HNSCC, regulation of GluCer is a possible viable pathway in that may be used in the treatment of HNSCC.

## 8. Therapies

While therapies targeting EGFR and human epidermal growth factor 2 (HER2) receptors are currently being used in the treatment of HNSCC (reviewed in [138]), clinical therapies specifically targeting SphK1 in HNSCC are scarce. PF-543 is a novel SphK inhibitor recently developed [139]. It is more than 100-fold more selective for SphK1 compared to SphK2, and was effective at suppressing endogenous S1P levels 10-fold with a proportional increase in sphingosine in HNSCC cell line 1483. PF-543 was also effective at reducing *ex vivo* S1P formation in human whole blood. Despite the striking change in cellular S1P and sphingosine levels, PF-543 had no effect on 1483 cell proliferation or survival. Although a single SphK1 inhibitor therapy agent was ineffective at reducing cell proliferation, combination therapies may prove to be more effective in targeting HNSCC. Whether this inhibitor will be of value as a therapy alone or in combination with other therapies is not known as we do not know the effect of this compound on tumorigenesis in animal models or in humans. Non-lipid SphK1 inhibitors such as SKI-I, SKI-II, and SK1-V have been used to decrease formation of S1P in mammary adenocarinoma xenograft models [140], but its effect in HNSCC has not been investigated. Likewise, use of FTY720 (fingolimod) is effective in reducing solid tumors of lung, pancreatic, prostate, breast, and colon cancers, but whether FTY720 is effective in reducing HNSCC tumors is unknown (reviewed in [141,142]). The development of a highly specific monoclonal S1P neutralizing antibody, LT1009 (human variant) provides another therapeutic alternative for the management of cancer [143,144]. This option is quite promising as LT1002 (mouse variant) inhibited tumor growth and angiogenesis in murine xenograft and allograft models. Similarly, the anti-S1P antibody has not been tested in HNSCC. However, it is a viable option because its use does not appear to be limited by toxic effects, and has shown promise in Phase I clinical studies [143]. 

Another potential therapy is safingol, a known PKC inhibitor with SphK-inhibiting properties [145]. It is important to note here that safingol’s effects are not specific to SphK1 as safingol functions as both a PKC inhibitor and also balances endogenous sphingolipid ceramide and S1P levels. Safingol induced cell rounding and detachment of SCC in monolayer cultures and this was a prerequisite for cell death [146]. Safingol increased Bim, decreased Bcl-xL and induced endonuclease G-mediated apoptosis; this occurred in a caspase-independent manner [146,147]. It is likely that future therapies involving Sphk1 will be used in conjunction with radiation or chemotherapy. This is best exemplified by a study that inhibited SphK1 and radiation to show that combination treatment improved radiation sensitivity of HNSCC xenografts in mice [35]. 

Ceramide-based therapies provide alternative treatment options for HNSCC (reviewed in [141]). As discussed previously, GEM/DOX treatment induces C18-ceramide and has, thus far, proved to be a viable option for treating metastatic and reoccurring HNSCC [105]. In addition, cationic ceramide analog L-threo-C6-pyridinium-ceramide-bromide treatment in combination with GEM was effective in preventing HNSCC tumor growth and progression *in vivo*, providing another viable alternative for HNSCC control [148]. 

Acid ceramidase, another sphingolipid metabolic enzyme, catalyzes the hydrolysis of ceramide to sphingosine. Acid ceramidase is over expressed in 70% of HNSCC [149] and may have a role in HNSCC treatment. Acid ceramidase inhibitor LCL204 was shown to sensitize HNSCC to Fas-induced apoptosis both *in vitro* and *in vivo* xenograph models [150]. Since the sphingolipid metabolism maintains the balance of bioactive lipids, this is not to say that these sphingolipid-related therapies do not affect SphK1 levels. 

Dietary nutrition is another way to modulate the SphK1/S1P axis, albeit this has not been demonstrated directly in HNSCC [151]. In prostate cancer, resveratrol, epigallocatechin gallate, and polyphenols from green tea or grapevine extract (vineatrol) suppressed SphK1 protein expression in prostate PC-3 cells, with the greatest effects seen one to three days after treatment. Not only did Sphk1 overexpression protect prostate cancer cells from green tea and wine polyphenol-induced death, but green tea and polyphenol treatment down-regulated SphK1 activity. This downregulation of SphK1 occurred in an ERK1/2/phospholipase D (PLD)-dependent manner. In addition, green tea polyphenol treatment resulted in decreased orthotopic tumor size, total tumor load, and metastasis. Concomitantly, there was a significant inhibition of SphK1 activity (>60%) and S1P content and significantly increased ceramide content. Thus, the authors propose that polyphenols directly regulate ERK1/2, in turn control PLD, which then manipulates the SphK1/S1P pathway to affect tumor growth in prostate cancer. These results are consistent with reports that also showed resveratrol modulates survival and apoptotic factors (including ceramide); these effects were mediated by MAPK and tyrosine kinases (reviewed in [152]). Natural inhibitors of SphK1 should be considered as a practical and feasible option for HNSCC therapy. 

## 9. Summary and Conclusions

In summary, SphK1 upregulation is associated with tumorigenesis and poor survival in HNSCC patients. The role of SphK1 in HNSCC is most likely due to increased S1P levels in tumor microenvironments and S1P interaction with various cell surface receptors. S1P has been shown to interact with various receptors, (*i.e.*, EGFR, TGFβ, S1PR), and this may explain how SphK1 regulates HNSCC. 

It cannot be excluded that modulations of SphK1 may be partly due to alterations of other sphingolipids and enzyme levels. Thus far, it seems that SphK1’s effects in HNSCC are S1P-dependent and whether SphK1 is capable of exerting S1P-independent effects remains to be elucidated. Additional work is required to fully understand the effect of SphK1 on proliferation, invasion and metastasis in HNSCC.

There have been many studies describing the relationships between SphK1 and cell surface receptors in HNSCC. However, the lack of similar studies makes it difficult to verify the findings. In addition, it is difficult to weave the current available published data together to produce a cohesive story because of the different cell models used. In addition, these studies focused on a wide array of pathways, making it difficult to relate one study to another, resulting in a large overview of SphK1 in HNSCC, at the expense of complete understanding of a single pathway. Furthermore, many studies have identified overexpression of SphK1 in HNSCC tumors and provided preliminary evidence of involved mechanisms, but follow-up studies verifying and explaining the existence of these pathways does not exist. Albeit, the multiple plausible mechanisms underlying SphK1 in HNSCC makes studying all these pathways is a big task as the amount of research needed is quite large. Research showing a casual relationship between SphK1 and metastasis, invasion and proliferation in an intact physiological model is warranted. Nevertheless, SphK1 remains a promising avenue for the treatment and therapy of HNSCC as research to date identifies SphK1 association with malignancy of HNSCC. Knockdown of SphK1 through *in vivo* delivery or SphK1 inhibition may prove to effective therapeutic strategies to increase sensitivity of HNSCC tumors to radiation or chemotherapy. A more complete understanding of SphK1 in HNSCC is required to initiate the development and utilization of therapies exploiting SphK1.
